# The effect of objective structured clinical examinations for nursing students

**DOI:** 10.1371/journal.pone.0286787

**Published:** 2023-06-09

**Authors:** Eun-Ho Ha, Eunju Lim

**Affiliations:** 1 Department of Nursing, Jungwon University, Chungbuk, Republic of Korea; 2 Red Cross College of Nursing, Chung-Ang University (CAU), Seoul, Korea; Qatar University, College of Health Sciences, QATAR

## Abstract

Twenty core nursing skills have been identified by the Korean Accreditation Board of Nursing Education. Proficiency in these skills is essential for all nursing professions, and many educational strategies exist to develop these skills in nursing students, including the Objective Structured Clinical Examination (OSCE). To date, no study on the effects of the OSCE on nursing education has been published. Therefore, we evaluated the effects of the OSCE on the core nursing skills of 207 pre-licensure nursing students in Korea. We measured the nursing students’ confidence, skills, and knowledge acquisition and retention. A one-way analysis of variance and Fisher’s least significant difference were used for data analysis. Among the four nursing areas (fall, transfusion, pre-operative, and post-operative), students demonstrated the highest confidence level scores in pre-operative nursing. On the OSCE, students scored the highest in transfusion nursing. Significant differences were found between prior knowledge, knowledge acquisition, and retention. Our findings confirm that the OSCE, after lectures and core nursing skill practice, improved the retention of nursing students’ knowledge. Therefore, this program can positively influence nursing students’ knowledge level, and implementing the OSCE can strengthen students’ clinical competency.

## Introduction

Core nursing skills are essential for the nursing profession [1]. Educational strategies to help nursing students achieve these skills should be well organized from the beginning of the nursing curriculum and managed as a continuing education component after graduation [2, 3]. The Korean Accreditation Board of Nursing Education (KABONE) has listed 20 core nursing skills that nursing students should master before graduation. The KABONE recommended that these skills be applied to the Clinical Performance Examination (CPX) [4].

The Objective Structured Clinical Examination (OSCE) is a clinical examination that reinforces practical competencies. It was first implemented in the mid-1970s to evaluate the clinical performance ability of medical students [5] and quickly became a method for the technical evaluation of nursing and healthcare-affiliated students [6–9].

The OSCE evaluates the skills of nursing students using an education method that translates classroom theory and in-class clinicals into clinical training [10]. As a result, educators have developed scenarios that reflect the learning objectives and content of the OSCE, arranged OSCE stations, and created virtual realities using a simulator or trained standardized patients [8, 11]. Using the OSCE, students are evaluated on basic core nursing skills and knowledge, patient and team-member communication skills, the education of patients and family members, patient assessment, and problem-solving [8, 12]. The OSCE checklist for evaluating students should consist of items that incorporate all aspects of nursing practice. Specifically, cognitive, affective, and psychomotor domains should be included to enhance the nursing profession [11, 13, 14].

The OSCE is an objective method for assessing students’ knowledge and clinical skills, as well as their interpersonal relationships, coping, critical thinking, and problem-solving skills [15]. The OSCE can quantify a student’s learning level and is an effective educational tool that allows self-reflection through immediate feedback on the student’s strengths and weaknesses [16, 17]. Furthermore, the OSCE has been shown to increase the motivation to learn, strengthen communication skills, and improve basic nursing skills, which consequently has been shown to promote confidence in nursing skills and learning satisfaction [16, 18, 19]. Students also reported experiencing positive feelings and believed the OSCE was a meaningful learning method [20]. The OSCE has also been reported to be a significant predictor for assessing students’ clinical competence before graduation and can be utilized during the semester and at the final examination [3]. In addition, the OSCE provides opportunities for nursing students to learn how to assess patients, communicate with patients, and solve nursing problems in actual clinical settings [16]. More importantly, experience gained during the OSCE process allows nursing students to easily adapt to the actual clinical field after graduation and may be essential for new nurses to gain confidence in their clinical performance in a real-world clinical setting [16, 21].

However, the immediate problems associated with the OSCE are the financial burden and lack of a skilled workforce [22]. In addition, students who experience anxiety, restlessness, and stress due to the OSCE may experience negative feelings, including distrust in the evaluation methods for nursing skills and uncertainty about the OSCE as a strategic learning method to assess students’ clinical competencies [10, 23]. Further, the psychological burden of learning and being evaluated on multiple skills simultaneously, and the inconsistency in the demonstrations across educators, may confuse students and lead them to view the OSCE as a waste of time [16]. The limited evidence on securing internal consistency, reliability, and validity for the OSCE checklists [24] may be an obstacle in expanding the OSCE, used as a license acquisition test, to the healthcare field.

From an academic perspective, the effect of the OSCE on nursing students’ knowledge has not been studied. Therefore, the current study examined the effects of learning while using the OSCE to measure students’ core nursing skills, knowledge acquisition and retention, and confidence. Moreover, the results of this study can serve as a baseline for nursing programs that are developing scenarios and checklists for OSCE learning.

## Materials and methods

### Research design

This study was a descriptive research study that examined the effects of the OSCE on the core nursing skills of nursing students.

### Participants

Participants included 207 (25 males, 182 females) pre-licensure nursing students enrolled in the CPX class at R College in Seoul, Republic of Korea. The 207 participants included the total number of students in the third year of nursing, which were automatically able to participate in this study because of the requirement to complete the CPX class before graduation. However, for the ethical consideration of the research participants, the purpose and method of the study were explained to all participants before the CPX class began. The intention to participate in the study was first verbally confirmed, and consent to participate was obtained. The sex and gender equity in research (SAGER) guidelines recommend that research be designed and conducted so that sex differences can be revealed in the results if human subjects are used and sex can be distinguished [25].

The CPX class, a mandatory course for pre-licensure nursing students in the second semester, focused on evaluating the 20 core nursing skills identified by the KABONE, including performance on the OSCE. During the course orientation, students were informed about the OSCE process and were provided information on the course’s study plans, objectives, and instructional methods. The students were also informed that completing the questionnaires for this study would not affect their grades, and there would be no penalty for not completing them.

### Questionnaires

#### Self-confidence

**Fig 1:**
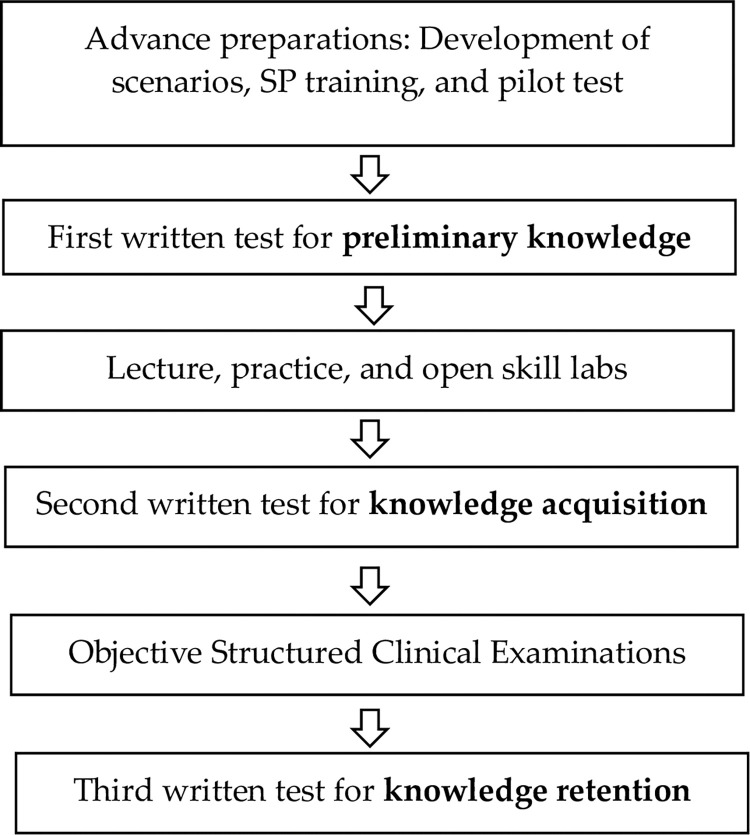
Flow chart outlining the procedure for assessing the nursing students’ preliminary knowledge, knowledge acquisition, and knowledge retention using questionnaires.

The researchers and other professionals developed a questionnaire measuring confidence in nursing care (Fig 1). This questionnaire included 16 questions across four categories, including situational awareness (four questions), nursing assessment (four questions), nursing intervention (four questions), and nursing evaluation (four questions) regarding fall, transfusion, pre-operative, and post-operative nursing. The response options for each question ranged from 1 (not at all confident) to 5 (very confident), with higher scores indicating greater self-confidence. The Cronbach’s alphas in this study were as follows: fall nursing, α = 0.86; transfusion nursing, α = 0.88; pre-operative nursing, α = 0.87; post-operative nursing, α = 0.87.

#### Prior knowledge and knowledge acquisition/retention

To measure students’ preliminary knowledge and acquisition and retention of knowledge in the four areas of nursing, a team of experts was created consisting of two Fundamentals of Nursing educators in the Fall Nursing course, two Pre- and Post-Operative Nursing educators in the Adult Nursing course, and two Transfusion Nursing educators in the Pediatric Nursing course (Fig 1). Following an explanation of the purpose and method of the study, the students were provided a 47-question questionnaire for evaluating their knowledge, which included questions regarding fall nursing (10 questions), pre-operative nursing (10 questions), post-operative nursing (15 questions), and transfusion nursing (12 questions). Each question was rated on a point scale, with higher points indicating a higher level of knowledge. The first evaluation of students’ preliminary knowledge was announced through the college’s homepage, which provided the curriculum of the four required areas of nursing for the students to study (Fig 1). The second evaluation of knowledge acquisition was conducted after the completion of individual or group self-learning for one week in the nursing school’s skill lab after lectures and practice (Fig 1). The third evaluation of knowledge retention was performed two weeks after the evaluation of clinical competency through the OSCE (Fig 1).

### Development of the OSCE scenarios and checklists

We formed a group of experts consisting of six nursing professors to evaluate students’ clinical performance. Among the 20 core nursing skills outlined by the KABONE, we excluded the following four skills from this evaluation: bowel enema, pulse oximetry and electrocardiogram, endotracheal suction, and cardiopulmonary resuscitation. Four areas of nursing (fall, transfusion, pre-operative, and post-operative) were classified encompassing the remaining 16 core nursing skills. Four unit managers from S General Hospital were invited to participate in this study to develop an effective checklist, along with scenarios that reflect the clinical situations of the four areas of nursing. The checklist for each category ranged from 0 to 2 points, with 2 = very good, 1 = moderate, and 0 = poor. Polit et al. [26] recommends that the content validity index (CVI) value measured by at least three experts should be .78 or higher. In order to validate the OSCE checklist based on the results of Bobos et al. [24] and Kolivand et al. [27], our group of experts found that the average CVI value was .93.

### Recruitment of standardized patients and training

Three scenarios were developed, Scenario 1 for fall nursing, Scenario 2 for pre- and post-operative nursing, and Scenario 3 for transfusion nursing. Three standardized patients from the R College Simulation Center were recruited (one for each scenario), including a 73-year-old woman for fall nursing, a 42-year-old woman with a history of a cesarean section for pre- and post-operative nursing, and a 38-year-old woman whose child had a history of hospitalization for transfusion nursing. Training materials from the R College Simulation Center were used to educate the standardized patients. Professors with experience training standardized patients were asked to provide instruction.

### Pilot test and inter-rater reliability

A pilot test was conducted to verify the simulation scenarios and checklists. This process helped identify ambiguous sentences in the simulation scenarios. Further, duplicate items on the checklists were modified and supplemented. To assess evaluator consistency, four evaluators from the four simulation areas participated in the pilot test. They provided ratings on the checklist simultaneously to determine the criteria for evaluation.

### Written test for prior knowledge and knowledge acquisition/retention

The first evaluation of students’ prior knowledge was conducted before the start of the CPX class. The educational content related to the four areas of nursing was announced through the college’s website. The second evaluation of knowledge acquisition was conducted at the end of a week of individual and group studies in the open clinical room, following four weeks of lectures and one week of clinicals. The third evaluation of knowledge retention was performed two weeks after the CPX class.

### Lecture and practice

Based on the 16 core nursing skills included in the questionnaires, four professionals from the four areas of nursing (fall, transfusion, pre-operative, and post-operative) designed a standardized lecture course. The 207 students were divided into two groups, each receiving eight hours of lectures a week for four weeks. Directly after the lectures, open skill labs were conducted to practice the 16 core nursing skills.

### Open skill labs

In order to provide opportunities to practice the 16 core nursing skills, skill labs were opened for students for one week following the lecture and skills practice. Four trained teaching assistants were available during these open skill labs to help students and examine their nursing skills.

### Objective Structured Clinical Examinations (OSCEs)

The four areas of nursing encompassing the 16 core nursing skills included in the questionnaires were evaluated by separating the students into pairs and rotating between the four OSCE stations. The time allotted for the evaluation was ten minutes in each area, and the time allotted for debriefing was 20 minutes in each area.

### Ethical considerations

This study obtained approval from the Institutional Review Board of Chung-Ang University. Participants were enrolled only after they provided written, informed consent. In addition, participants were informed that they could choose not to participate in the study at any time without any personal disadvantage or penalty. The collected data were maintained in the researchers’ private office, which was kept locked to ensure confidentiality.

### Data analyses

All statistical analyses were performed using SPSS version 20.0 (SPSS Inc., Chicago, IL). Descriptive statistical analysis was used for the general characteristics of the participants, confidence in nursing, and core nursing skills evaluation. For three or more time points or replicate conditions, a repeated measures analysis of variance (ANOVA) for independent samples should be used. Therefore, continuous outcome and categorical independent variables are required. ANOVAs evaluate correlations within variables and have the advantage of increasing the accuracy of research because even small changes can be captured [28]. Therefore, the evaluation of preliminary knowledge (first), knowledge acquisition (second), and knowledge retention (third) for four independent variables (falls, transfusion, pre- and post-operative nursing) was performed using a repeated one-way ANOVA.

A Mauchly’s test of sphericity was used to validate the hypothesis of sphericity, and the results of the interpretive effect assay were used. A Fisher’s Least Significant Difference (LSD) was used for post-hoc analyses.

## Results

### General characteristics of participants

The average age of the participants was 23.6 years, where 107 (51.7%) participants were under 23 years, and 100 (48.3%) were 23 years or older (Table 1).

**Table 1:** General characteristics of participants (*N* = 207).

Variable	Category	*n*	Percent (%)
Age (*M* = 23.6 years, *SD =* 3.79)	< 23	107	51.7
	≥ 23	100	48.3
Sex	Males	25	12.1
	Females	182	87.9
Type of prior learning	Self-study	116	56.0
	Group study	89	43.0
	No study	2	1.0
Open Skill Labs	Very helpful	136	65.7
	Slightly helpful	64	30.9
	Not helpful at all	7	3.4
Awareness of 20 Core Nursing Skills	Know well	98	47.3
	Know slightly	108	52.2
	Do not know	1	0.5
Awareness of the OSCE	Know well	140	67.6
	Know slightly	66	31.9
	Do not know	1	0.5
OSCE	Very helpful in CA	175	84.5
	Slightly helpful in CA	32	15.5
	Not helpful at all	0	0.0
The OSCE is necessary for CC	Yes	180	87.0
	Maybe	27	13.0
	No	0	0.0

OSCE = Objective Structured Clinical Examination; CA = Clinical Adaptation; CC = Clinical Competency; M = Mean; SD = Standard Deviation

### Level of self-confidence and the OSCE

The highest self-confidence score for patient nursing was in pre-operative nursing, with a mean score of 18.21 (*SD* = 2.03) (Table 2). The highest situational awareness score was also in pre-operative nursing, with a mean score of 4.63 (*SD* = 0.55). The highest assessment score was in fall nursing, with a mean score of 4.55 (*SD* = 0.62) (Table 2). The highest nursing intervention score was in pre-operative nursing, with a mean score of 4.59 (*SD* = 0.58). The highest nursing evaluation score was in fall nursing, with a mean score of 4.45 (SD = 0.67). For the OSCE, the highest score was in transfusion nursing, with a mean score of 1.74 (SD = 0.17) (Table 2).

**Table 2:** Level of self-confidence and the objective structured clinical examination (*N* = 207).

	**Fall Nursing**	**Transfusion Nursing**	**Pre-operative Nursing**	**Post-operative Nursing**	**Range**
	M (SD)	M (SD)	M (SD)	M (SD)	
Self-Confidence	18.15 (2.08)	17.32 (2.55)	18.21 (2.03)	18.07 (2.14)	4–20
Situational awareness	4.58 (0.60)	4.35 (0.77)	4.63 (0.55)	4.62 (0.54)	1–5
Assessment	4.55 (0.62)	4.30 (0.74)	4.54 (0.61)	4.52 (0.63)	1–5
Nursing intervention	4.57 (0.59)	4.34 (0.74)	4.59 (0.58)	4.52 (0.67)	1–5
Nursing evaluation	4.45 (0.67)	4.33 (0.71)	4.44 (0.64)	4.41 (0.68)	1–5
OSCE	1.69 (0.17)	1.74 (0.17)	1.64 (0.24)	1.68 (0.18)	0–2

*M =* mean; *SD =* standard deviation; OSCE = Objective Structured Clinical Examination

### Effect of OSCE on knowledge acquisition and retention

The mean preliminary knowledge (first) score for fall nursing was 5.79 (*SD* = 0.09), and following lectures and practice for knowledge acquisition (second), the mean significantly decreased to 5.49 (*SD* = 0.10) (Table 3). However, after the OSCE for knowledge retention (third), the mean score significantly increased to 5.92 (*SD* = 0.10, *p* < 0.001). The mean preliminary knowledge score for transfusion nursing was 7.41 (*SD* = 0.12), and after the lecture and practice for knowledge acquisition, the mean score increased to 7.84 (*SD* = 0.11) (Table 3). Additionally, following the OSCE for knowledge retention, the mean score significantly increased to 8.31 (*SD* = 0.10, *p* < 0.001). The mean preliminary knowledge score for post-operative nursing was 11.44 (*SD* = 0.10), and the knowledge acquisition score was increased to 11.70 (*SD* = 0.11) (Table 3). Moreover, after the OSCE for knowledge retention, the mean score significantly increased to 12.15 (*SD* = 0.10; *p* < 0.001) (Table 3).

**Table 3:** Effect of the OSCE on knowledge acquisition and retention (*N* = 207).

**Variable (range)**	**Preliminary Knowledge**^**a**^ **(First)**	**Knowledge Acquisition**^**b**^ **(Second)**	**Knowledge Retention**^**c**^ **(Third)**	***F***	***p***	**Multiple Comparison** ^*^
	*M* (*SD*)	*M* (*SD*)	*M* (*SD*)			
Fall nursing (0–10)	5.79 (0.09)	5.49 (0.10)	5.92 (0.10)	8.06	< 0.001	a > b, b < c
Transfusion nursing (0–12)	7.41 (0.12)	7.84 (0.11)	8.31 (0.10)	22.52	< 0.001	a < b < c
Pre-operative nursing (0–10)	7.92 (0.08)	7.22 (0.07)	7.75 (0.06)	36.86	< 0.001	a > b, b < c
Post-operative nursing (0–15)	11.44 (0.10)	11.70 (0.11)	12.15 (0.10)	18.74	< 0.001	a < b < c

*M =* mean; *SD =* standard deviation

*Fisher’s Least Significant Difference

## Discussion

This study was conducted to assess the effectiveness of the implementation of the OSCE by examining the level of core nursing skills required for Korean nursing students before graduation and the changes in the level of knowledge related to these core nursing skills. This information would be useful for educators to ascertain which course elements have the most optimal effect on student progress.

In this study, all participants reported that the OSCE was “very helpful or slightly helpful” for students adapting to clinicals. Other research has examined the perceptions of the OSCE of nursing students who were in the process of obtaining their bachelor’s degree using thematic content analysis [29]. This research reported that the OSCE was meaningful in preparing for clinical practice, similar to our current study. Additionally, in response to the question regarding the OSCE being necessary for clinical competency, all participants in the previously published study stated that it “is helpful,” which is similar to findings from our study. Although the OSCE may create additional stress for students, it is recognized as an objective evaluation method [30]. Therefore, the OSCE has a low risk of inter-evaluator prejudice because of its high objectivity and high level of reliability and validity [18].

Among the four areas of nursing (fall, transfusion, pre-operative, and post-operative), the participants in this study showed the highest level of confidence in pre-operative nursing and the lowest level of confidence in transfusion nursing. These results have not been previously reported, as no comparable studies have been conducted. However, these results may be due to the lack of proper awareness of patients’ situations, the burden of prompt reporting, and the difficulty in communicating with the caregivers of patients in transfusion nursing.

Across all four areas of nursing, situational awareness, compared to assessment, nursing intervention, and evaluation, showed a higher score in the self-confidence assessment. According to Miller [31], cognition, compared to performance, a psychomotor function, is a subordinate concept that is easier to accomplish with relatively higher confidence. In this study, situational awareness pertains to cognition, whereas assessment, nursing intervention, and evaluation pertain to performance. Thus, the current results resemble Miller’s Pyramid Model [31]. Therefore, it is necessary to develop a module that harmoniously improves cognitive nursing skills with psychomotor nursing skills.

Regarding the level of achievement in the OSCE, the transfusion nursing performance level was the highest, and the post-operative performance level was the lowest. These results suggest that nursing students find it more difficult to perform post-operative nursing, which consists of skin tests and indwelling catheter insertion, rather than transfusion nursing, which consists of taking vital signs and oxygen therapy. According to the KABONE [4], vital signs and oxygen therapy are low-level skills, as they are relatively easy compared to skin tests and indwelling catheter insertion, which are considered high-level skills. Therefore, it is important to consider the placement of the OSCE stations so that the difficulty levels of the core nursing skills are evenly distributed.

In the case of transfusion and post-operative nursing, the second and third knowledge evaluations showed a significant and continuous increase in knowledge, which confirms that the OSCE can be effective in retaining the level of nursing knowledge. However, in a report from the National Council of State Boards of Nursing (NCSBN), the level of knowledge after the OSCE was found to significantly decrease from the baseline [32], showing a different result from our current findings. Meanwhile, our study conducted the second knowledge evaluation after lectures and core nursing skill practice and showed significantly lower results in fall and pre-operative nursing compared to the preliminary knowledge evaluation. However, at the time of the third evaluation, the level of knowledge increased, which confirms that lectures and core nursing skill practice should run parallel with the OSCE as it is more effective in improving and retaining knowledge. These results are consistent with those of a study conducted by Kardong-Edgren et al., which reported that opportunities for the OSCE could improve nursing knowledge, cognitive reasoning, and critical thinking and help with nursing performance and communication skills [33].

Core nursing skills are the main requirements for registered nurses to perform various nursing duties in clinical settings [4, 34]. This study confirmed that the OSCE, in addition to lectures and core nursing skill practice, improved knowledge retention. These methods can positively affect the level of knowledge in nursing students, and implementing the OSCE can strengthen students’ clinical competency.

## Conclusions

This study was significant because it provided a valuable suggestion for utilizing the OSCE. Nursing students that participated in this study appreciated the benefit of the OSCE in clinical adaptations and viewed it as a necessary part of the curriculum. In all four areas of the OSCE, the participants showed a higher level of confidence in situational awareness than in any other subcategories. Therefore, an OSCE-based curriculum allows learners to retain knowledge effectively.

In addition to the review of core nursing skills and knowledge before graduation, we suggest follow-ups to evaluate how recently-graduated nurses demonstrate their core nursing skills in a real clinical setting. Such a longitudinal survey would provide information on how student learning outcomes are related to nursing practices.

Although these results are informative, several limitations of this study should be considered. First, as the study sample included students from only one nursing college, the results may not apply to other healthcare professions with different experiences. Second, the OSCE stations encompassing the 16 core nursing skills used in this study were developed by researchers and the KABONE in South Korea. Therefore, these stations may not be suitable for generalization to larger populations or those in other countries. Third, this is a descriptive study that investigated the effect of OSCE learning in a single group without a control group. Therefore, the learning effect of the OSCE cannot be evaluated. Fourth, we cannot rule out that there were limitations in securing the reliability of the OSCE checklist, which could affect the results of the study. Overall, this study showed an improvement in the retention of nursing students’ knowledge before graduation using the OSCE after lectures and core nursing skill practice. We suggest follow-up evaluations using the OSCE for the continued education of recently-graduated nurses to maintain proficiency in these core nursing skills.

## Supporting information

S1 Dataset (SAV)

S1 File (DOCX)
