# Supplementary material for: The effect of objective structured clinical examinations for nursing students
Source: PLoS One. 2023 Jun 9;18(6):e0286787. doi: 10.1371/journal.pone.0286787 (PMC10256199; doi:10.1371/journal.pone.0286787)
Supplement: S1 File — (DOCX) [file pone.0286787.s002.docx]

Supporting information: The Effect of Objective Structured Clinical Examinations for Nursing Students submitted with DOI <https://doi.org/10.5061/dryad.x95x69ppv> or <https://datadryad.org/stash/share/RQawdE6kKGufCbzJGCKvpplJ9BoKYyK65PxFxaXhiAE>.
